# Supplementary material for: Challenges and enablers in measles vaccination implementation in Ethiopia: Insights from a qualitative study
Source: PLOS Glob Public Health. 2025 Jul 7;5(7):e0004859. doi: 10.1371/journal.pgph.0004859 (PMC12233235; doi:10.1371/journal.pgph.0004859)
Supplement: S1 Data — (DOCX) [file pgph.0004859.s001.docx]

| **S1 Data: Mothers/caregivers in-depth interview guide targeted for assessing reasons for measles vaccination dropout** |
| --- |
| **Introduction**  Good morning/afternoon. My name is _______________________________ and I would like to respectfully ask for your help in answering the questions in this interview. Our goal is to help improve the vaccination program in general. I know you are busy, so we will interview you for only a few minutes. Your participation is completely voluntary and anonymous. Would you be willing to answer these questions?  Thank you very much. |
| **House Hold Demographic Data**  Date of Interview___/_____/_________ Interview start time:_______________  Region__________ Zone______________ Woreda_________ Kebele________ House No______  Name of Respondent_________________________, Age____ Sex: M F Religion____________, Primary Language____________ |
| Marital Status: **A**. Single **B**. Married **C**. Divorced/separated **D**. Widowed,  Respondent’s Relation to child____________________ Number of HH members__________  Can you read and write? **A**. Cannot read or write, **B**. Read only **C**. Read and write  Formal education: **A**. No formal education, **B**. Did not complete 1^0^, **C**. Completed 1^0^ **D**. Secondary Completed **E**. 2^0^ +  Spouse’s Level of Education: **A.** No formal education, **B.** Did not complete 1^0^, **C**. Completed 1^0^ **D**. Secondary Completed **E.** 2^0^ +  What do you do for a living?  A. Housewife B. Laborer C. Farming D. Self-employed E. Student F. Merchant G. Employee H. Other Specify: __________________  Spouse’s Occupation: A. Housewife B. Laborer C. Farming D. Self-employed E. Student F. Merchant G. Employee H. Other Specify: __________________  Are you Head of the HH? A. Yes B. No  What is your role in the community? ____________________________________ |
| Family Income (Annual) Birr. _________ |

**In-Depth Interview Questions**

1. Do you have access ***at least every week*** to a phone radio or television?
2. Do you know of a health post/facility that you can use in this area? How far is it or how long does it take you to reach it?
3. Do you need to ask for permission if you need to travel to a health post/facility? Who would you need to ask for permission? ***Why didn’t you have the autonomy?***
4. When did you last travel to a health post/facility? The last time you visited a health post/facility, how did you travel and how much did it cost you? How expensive was the cost of transportation?
5. What was the main reason for your last visit to a health post/facility and was the health facility open when you arrived?
6. Did you ever have ANC, delivery or PNC during your last pregnancy?
7. Do you have any sons or daughters to whom you have given birth and who are now living with you? Are you a guardian or caretaker of children you have not given birth to?
8. Have you ever heard of vaccinations and where have you heard from? What messages have you ever heard about vaccination or vaccines?
9. What benefits can a child get from immunization? Can you mention some of the vaccines or diseases prevented by vaccination? I f measles is not mentioned, probe for it.
10. In your opinion, who should get the vaccination provided in your area?
11. When do you think a child should ***first*** be vaccinated and how many times should a child get vaccines? How long after birth do you think a child should complete his/her immunization schedule?
12. Do you know of a place where a child can be immunized? Please tell me all the places where a child can be immunized?
13. Have you ever discussed immunizations with people around you and which kind of people are in favor of immunizing children?
14. Do you believe that all children under the age of one should receive routine vaccines?
15. Who usually makes ***decisions*** about vaccinating your children?
16. Do you have a card or other document where your child’s vaccinations are written down that I can see? If no card, why don't you have a vaccination card for child?
17. Why was your child not vaccinated or did not complete all vaccination before his/her first birthday? Probe both providers and demand side barriers.
18. Thinking about the immunization services provided at the health institutions or outreaches that you are familiar with, as well as your experiences with the health workers who provide immunization services, how satisfied are you about the quality of the services? Rate in scale.
19. Which part of the service makes you satisfied or dissatisfied: health workers’ reception, advice and waiting time?
20. Would you ever disseminate messages about the benefits of vaccination to your friends and neighbors and would you send the children of your friends, relatives, or neighbors for vaccination if you knew their child was not vaccinated?

**Child Immunization History**

| Child’s Date of Birth | - DAY MONTH YEAR: ____/___/____ |
| --- | --- |
| BCG | - DAY MONTH YEAR: ____/___/____ - CARD SHOWS VACCINE, BUT NO DATE - NOT GIVEN |
| ORAL POLIO VACCINE (OPV) 0 (BIRTH DOSE) | - DAY MONTH YEAR: ____/___/____ - CARD SHOWS VACCINE, BUT NO DATE - NOT GIVEN |
| ORAL POLIO VACCINE (OPV) 1 | - DAY MONTH YEAR: ____/___/____ - CARD SHOWS VACCINE, BUT NO DATE - NOT GIVEN |
| ORAL POLIO VACCINE (OPV) 2 | - DAY MONTH YEAR: ____/___/____ - CARD SHOWS VACCINE, BUT NO DATE - NOT GIVEN |
| ORAL POLIO VACCINE (OPV) 3 | - DAY MONTH YEAR: ____/___/____ - CARD SHOWS VACCINE, BUT NO DATE - NOT GIVEN |
| IPV | - DAY MONTH YEAR: ____/___/____ - CARD SHOWS VACCINE, BUT NO DATE - NOT GIVEN |
| PENTAVALENT 1 | - DAY MONTH YEAR: ____/___/____ - CARD SHOWS VACCINE, BUT NO DATE - NOT GIVEN |
| PENTAVALENT 2 | - DAY MONTH YEAR: ____/___/____ - CARD SHOWS VACCINE, BUT NO DATE - NOT GIVEN |
| PENTAVALENT 3 | - DAY MONTH YEAR: ____/___/____ - CARD SHOWS VACCINE, BUT NO DATE - NOT GIVEN |
| PNEUMOCOCCAL 1 | - DAY MONTH YEAR: ____/___/____ - CARD SHOWS VACCINE, BUT NO DATE - NOT GIVEN |
| PNEUMOCOCCAL 2 | - DAY MONTH YEAR: ____/___/____ - CARD SHOWS VACCINE, BUT NO DATE - NOT GIVEN |
| PNEUMOCOCCAL 3 | - DAY MONTH YEAR: ____/___/____ - CARD SHOWS VACCINE, BUT NO DATE - NOT GIVEN |
| ROTAVIRUS 1 | - DAY MONTH YEAR: ____/___/____ - CARD SHOWS VACCINE, BUT NO DATE - NOT GIVEN |
| ROTAVIRUS 2 | - DAY MONTH YEAR: ____/___/____ - CARD SHOWS VACCINE, BUT NO DATE - NOT GIVEN |
| MEASLES1 (MCV1) | - DAY MONTH YEAR: ____/___/____ - CARD SHOWS VACCINE, BUT NO DATE - NOT GIVEN |
| MEASLES2 ( MCV2) | - DAY MONTH YEAR: ____/___/____ - CARD SHOWS VACCINE, BUT NO DATE - NOT GIVEN |

**PROBE THE FOLLOWING FOR REASONS FOR NONE OR UNDER IMMUNIZATION (Q. 17)**

**BLOCK A: REASONS RELATED TO THE HEALTH WORKERS**

A. The Health worker said that the child was not eligible for vaccination

B. The health worker who saw us did not tell me about vaccinating the child

C. The health worker said that the child could not be vaccinated because s/he was sick

**BLOCK B: IF BECAUSE OF ILLNESS**

A. Minor illnesses such as mild fever, cold, cough, or diarrhea

B. Major illnesses requiring admission, such as severe pneumonia or severe malaria

C. Child is taking medications: Write down generic name ______________

D. Other Specify:

**BLOCK C: REASONS RELATED TO THE CAREGIVER**

1. I thought my child has completed all his scheduled immunization.
2. The last time the child was vaccinated, he/she got sick or had a reaction.
3. My religion or superstition doesn't permit vaccination, or I don't believe in vaccines
4. don't trust the health workers/the vaccines in this health facility
5. I forgot to take my child to the vaccination area
6. I didn't have time to wait for vaccination
7. Fear of loss of earning
8. Afraid of health worker because I missed an appointment
9. I don’t believe child need vaccination to grow or become healthy
10. I am poor and don’t look well-dressed to go out to public places
11. Other Specify: _____________________

**BLOCK D: REASONS RELATED TO THE HEALTH FACILITY (LOGISTICS & ORGANIZATION)**

1. Health facility is too far
2. There were no vaccines in the health facility;
3. There were no syringes or other vaccination supplies;
4. The vaccination day was not convenient for me
5. The health facility is usually closed
6. Frequent session interruptions
7. The person in charge of vaccinations is usually not available or punctual
8. There would have been a long waiting time
9. The staff treated me badly
10. Other Specify:

**PROBE THE FOLLOWING FOR REASONS FOR NONE OR UNDER IMMUNIZATION (Q. 18)**

1. The quality immunization services being provided at the health institution
2. Health workers involved in providing immunizations services are receptive.
3. Availability of health worker at the time of an appointment for immunizations.
4. Waiting time for immunizations.
5. Health care providers explaining why I should immunize my child.
6. Health care providers treats in a very friendly and courteous manner
7. Health care providers explain when I should return for the next immunizations.

| **Appendix 2: Focus group discussion (FGD) guide for mothers/caregivers of children aged 12-59 months** |
| --- |
| **Introduction**  Good morning/afternoon. My name is _______________________________ and I would like to respectfully ask for your help in participating in this discussion. Our goal is to help improve the vaccination program in general. I know you are busy, so we will interview you for only a few minutes. Your participation is completely voluntary and anonymous. Would you be willing to participate?  Thank you very much.  **Date of interview:___/_____/_____ Interview start time:________________**  **County/District: __________________________ Area: _________________________**  **Name of interviewer: ______________________ Supervisor: _____________________**  **Number of caregivers in the focus group: _____________________________________**  **FGD guide Questions** |

1. Do you know of a health post/facility that you can use in this area? How far is it or how long does it take you to reach it?
2. When did you last travel to a health post/facility? The last time you visited a health post/facility, how did you travel and how much did it cost you? How expensive was the cost of transportation?
3. What was the main reason for your last visit to a health post/facility and was the health facility open when you arrived?
4. Do you have any sons or daughters to whom you have given birth and who are now living with you? Are you a guardian or caretaker of children you have not given birth to?
5. Have you ever heard of vaccinations and where have you heard from? What messages have you ever heard about vaccination or vaccines?
6. What benefits can a child get from immunization? Can you mention some of the vaccines or diseases prevented by vaccination? If measles is not mentioned, probe for it.
7. In your opinion, who should get the vaccination provided in your in your area?
8. When do you think a child should ***first*** be vaccinated and how many times should a child get vaccines? How long after birth do you think a child should complete his/her immunization schedule?
9. Do you know of a place where a child can be immunized? Please tell me all the places where a child can be immunized?
10. Have you ever discussed immunizations with people around you and which kind of people are in favor of immunizing children?
11. Would you ever disseminate messages about the benefits of vaccination to your friends and neighbors and would you send the children of your friends, relatives, or neighbors for vaccination if you knew their child was not vaccinated?
12. Do you believe that all children under the age of one should receive routine vaccines?
13. Why were some children are not vaccinated or did not complete all vaccination before his/her first birthday…e.g measles vaccine? Probe both providers and demand side barriers.

List all the barriers, then use ranking table to prioritize/rank

1. Do you have any questions for us anything you want to add?

**PROBE THE FOLLOWING FOR REASONS FOR NONE OR UNDER IMMUNIZATION (Q. 13)**

**BLOCK A: REASONS RELATED TO THE HEALTH WORKERS**

A. The Health worker said that the child was not eligible for vaccination

B. The health worker who saw us did not tell me about vaccinating the child

C. The health worker said that the child could not be vaccinated because s/he was sick

**BLOCK B: IF BECAUSE OF ILLNESS**

A. Minor illnesses such as mild fever, cold, cough, or diarrhea

B. Major illnesses requiring admission, such as severe pneumonia or severe malaria

C. Child is taking medications: Write down generic name ______________

D. Other Specify:

**BLOCK C: REASONS RELATED TO THE CAREGIVER**

1. I thought my child has completed all his scheduled immunization.
2. The last time the child was vaccinated, he/she got sick or had a reaction.
3. My religion or superstition doesn't permit vaccination, or I don't believe in vaccines
4. don't trust the health workers/the vaccines in this health facility
5. I forgot to take my child to the vaccination area
6. I didn't have time to wait for vaccination
7. Fear of loss of earning
8. Afraid of health worker because I missed an appointment
9. I don’t believe child need vaccination to grow or become healthy
10. I am poor and don’t look well-dressed to go out to public places
11. Other Specify: _____________________

**BLOCK D: REASONS RELATED TO THE HEALTH FACILITY (LOGISTICS & ORGANIZATION)**

1. Health facility is too far
2. There were no vaccines in the health facility;
3. There were no syringes or other vaccination supplies;
4. The vaccination day was not convenient for me
5. The health facility is usually closed
6. Frequent session interruptions
7. The person in charge of vaccinations is usually not available or punctual
8. There would have been a long waiting time
9. The staff treated me badly
10. Other Specify:

**PROBE THE FOLLOWING FOR REASONS FOR NONE OR UNDER IMMUNIZATION**

1. The quality immunization services being provided at the health institution
2. Health workers involved in providing immunizations services are receptive.
3. Availability of health worker at the time of an appointment for immunizations.
4. Waiting time for immunizations.
5. Health care providers explaining why I should immunize my child.
6. Health care providers treats in a very friendly and courteous manner
7. Health care providers explain when I should return for the next immunizations

**Appendix 3: Exit interview guides for mothers/caregivers of children 12-23 months at health facilities**

The child appears to be <24 months

1. Yes CONTINUE, 2. No **THANK THE PERSON AND MOVE ON TO THE NEXT**

Good morning/afternoon. My name is _______________________________ and I would like to respectfully ask for your help in answering the questions in this survey. Our goal is to help improve the vaccination program in general. I know you are busy, so we will interview you for only a few minutes. Your participation is completely voluntary and anonymous. Would you be willing to answer these questions?

Thank you very much.

**Date of interview______________________Interview start time:**

**Name of interviewer: __________________ Supervisor: _______________**

**Name of health facility: __________________**

**Region _______________ Zone_____________________ Woreda _________________**

**Day |__|__| Month |___|___| Year |___|___| Hour |__|__| Minutes |___|___|**

**SECTION 1: DATA ON THE CHILD:** (IN CASE OF MORE THAN ONE CHILD, CHOOSE THE YOUNGEST CHILD)

1. Date of birth Day |___|___| Month |___|___| Year |___|___|

2. Sex or Gender of child 1. Male 2. Female

3. Why did you bring the child to this health care facility today? **(DO NOT READ OUT THE CHOICES)**

A. For medical consultation (child is sick), B. For vaccination, C. Healthy child visit or growth/ check-up

D. Child is only accompanying (not for treatment, vaccination), E. Hospitalization (child was admitted)

F. Other Specify: ____________________

4. Marital Status: 1. Single 2. Married 3. Divorced/separated 4. Widowed,

5. What is your relationship to the child?

A. Mother, B. Father, C. Grandparent, D. Brother/sister, G. Other Specify: _______

6. Can you read and write? A. Cannot read or write B. Read only C. Read and write

7. Level of formal education

A. No formal education, B. Did not complete 1^0^, C. Completed 1^0^  D. Secondary E. secondary +

8. What do you do for a living?

A. Housewife B. Laborer C. Farming D. Self-employed

E. Student F. Merchant G. Employee H. Other Specify: __________________

9. By what means of transportation do you usually come to this facility?

A. Walk B. Animal C. Motorcycle D. Car,

E. Bus F. Taxi F. Other Specify: ____________________

10. How long does it take you to get here? _____Hours _____Minutes

11. Have you heard or seen messages on vaccination in the last three month?

A. Yes **CONTINUE WITH QUESTION 12,** B. No **SKIP TO QUESTION 13**

12. Where/how did you hear or see the message? **(DO NOT READ OUT THE OPTIONS. circle ALL THAT APPLY)**

A. Radio, B. Television, C. Newspaper, D. Health facility, E. Telephone message, F. Facebook or internet

G. Children's school, H. Place of worship, I. During home visit by HEW/health outreaches

J. Community meetings, K. Other Specify: ____________________

13a. Do you know which vaccines your child needs?

A. Yes B. No C. Not sure

If YES --> 13b. Do you know WHEN the vaccines should be given?

A. Yes B. No C. Not sure

14. Has this child ever been vaccinated?

A. Yes B. No

If NO --> 14b. If no, why not? (**Circle ALL THAT APPLY)**

1. Not aware of the necessity of vaccination B. Not aware of the session site
2. Not aware of the session day D. Went on wrong day
3. Vaccination site is far
4. Husband or decision maker not in favor of vaccination G. Fear of fever or AEFI
5. Religious reason I. Child is sick
6. Fear of loss of earning K. Necessary vaccines/supplies unavailable
7. Other __________

15. Have you ever requested vaccination service for this child and been refused?

A. Yes, B. No **SKIP TO QUESTION 17**

16. If so, why didn't they vaccinate the child?

A. HW said it couldn't be done because the child was sick, B. There were no vaccines, or other supply

C. It was not a vaccination day, D. Vaccination post was closed,

E. The vaccinator was not there, F. Vaccination card/passport not with me

G. The hours for vaccination are limited H. HW said this is not my catchment

I. Other Specify: ____________________

17. In your home, who primarily makes the decision to vaccinate the children

A. Father, B. Mother, C. Other relatives, D. Consensus of father and mother, E. Other Specify: _________

18. Does your child have a vaccination card/health passport?

A. Yes, and I have it with me **GO TO QUESTION 20**,

B. Yes, but I do not have it with me **GO TO QUESTION 19a** C. No **GO TO QUESTION 19b**

19a. Could you tell us why you do not have the vaccination card/health passport with you today?

A. I forgot to bring it, B. I didn't know it was important to bring it,

C. I lost it SKIP TO QUESTION 22 D. The card/health passport has been damaged,

F. I have never been given one **SKIP TO QUESTION 23**

G. Because vaccination was not the reason for this visit, **SKIP TO QUESTION 21**

H. Other Specify: ____________________

19b. Why don't you have a vaccination passport?

A. I lost it SKIP TO QUESTION 22, B. I have never been given one SKIP TO QUESTION 23,

C. I don't know, D. Other Specify: ____________________

20. Request and examine the child's vaccination card/health passport or temporary vaccination document to

fill out the following table.

| **Vaccines** | **Date Administered as Written on vaccination Card** | | | |
| --- | --- | --- | --- | --- |
|  | **Doze 0** | **Dose 1** | **Dose 2** | **Dose 3** |
| **BCG** |  | ***___\|___\|___*** |  |  |
| **Oral Polio** | ***___\|___\|___*** | ***___\|___\|___*** | ***___\|___\|___*** | ***___\|___\|___*** |
| **IPV** |  | ***___\|___\|___*** |  |  |
| **DTP-HepB-Hib** |  | ***___\|___\|___*** | ***___\|___\|___*** | ***___\|___\|___*** |
| **Rotavirus** |  | ***___\|___\|___*** | ***___\|___\|___*** |  |
| **PCV** |  | ***___\|___\|___*** | ***___\|___\|___*** | ***___\|___\|___*** |
| **MCV1** |  | **___\|___\|___** | **___\|___\|___** | **___\|___\|___** |
|  |  |  |  |  |

21. Have you ever lost a vaccination card/health passport for this child?

A. Yes, B. No **SKIP TO QUESTION 23**

22. Did you encounter difficulty getting it replaced?

A. Yes, B. No

23. Could you tell me what purpose the vaccination card/health passport serves?

**(DO NOT READ OUT THE OPTIONS. CHECK ALL THAT APPLY)**

A. To know what vaccines the child has, and the missing ones, B. Birth certificate and/or identification

C. Overall health record and growth monitoring, D. Record and remind for return visit dates

E. Other, F. Don't Know/No Response Specify: ____________________

24. During today's visit, did the personnel/staff ask you for the child's vaccination card/health passport?

A. Yes **SKIP TO QUESTION 26,** B. No **SKIP TO QUESTION 25**

25. If No, did they ask for the vaccination status of the child?

A. Yes, B. No

26. Was your child vaccinated here today?

A. Yes **SKIP TO QUESTION 29,** B. No

**27. Why was your child not vaccinated today?**

**BLOCK A: REASONS RELATED TO THE HEALTH WORKERS**

A. The Health worker said that the child was not eligible for vaccination today

B. The health worker who saw us did not tell me about vaccinating the child today

C. The health worker said that the child could not be vaccinated because s/he was sick

**IF BECAUSE OF ILLNESS, WHAT TYPE OF DISEASE OR TREATMENT DID THE CHILD RECEIVE TODAY?**

A. Minor illnesses such as mild fever, cold, cough, or diarrhea

B. Major illnesses requiring admission, such as severe pneumonia or severe malaria

C. Child is taking medications: Write down generic name ______________

D. Other Specify: ____________________

**BLOCK C: REASONS RELATED TO THE CAREGIVER**

1. I thought my child has completed all his scheduled immunization.
2. The last time the child was vaccinated, he/she got sick or had a reaction.
3. My religion or superstition doesn't permit vaccination, or I don't believe in vaccines
4. don't trust the health workers/the vaccines in this health facility
5. I forgot to take my child to the vaccination area
6. I didn't have time to wait for vaccination
7. Fear of loss of earning
8. Afraid of health worker because I missed an appointment
9. I don’t believe child need vaccination to grow or become healthy
10. I am poor and don’t look well-dressed to go out to public places
11. Other Specify: _____________________

**BLOCK D: REASONS RELATED TO THE HEALTH FACILITY (LOGISTICS & ORGANIZATION)**

1. Health facility is too far
2. There were no vaccines in the health facility;
3. There were no syringes or other vaccination supplies;
4. The vaccination day was not convenient for me
5. The health facility is usually closed
6. Frequent session interruptions
7. The person in charge of vaccinations is usually not available or punctual
8. There would have been a long waiting time
9. The staff treated me badly
10. Other Specify:

28. If the child was eligible for vaccination but was not vaccinated today, did the health worker refer you to

or inform you where you can receive the missing vaccine doses?

A. Yes B. No: **SKIP TO QUESTION 39**

29. How long did you wait today for your child to be vaccinated? Hours: _____ Minutes: ______

30. Today after vaccination what key messages did the health worker tell you?

A. Vaccines they gave the child?

B. Date of the next vaccination appointment?

C. The reactions or side effects that can occur following vaccination?

31. If so, what did they mention? (choose all that apply)

1. Pain at injection site Vomiting
2. Fever
3. Rash
4. Diarrhea
5. Vomiting
6. Other __________________

32. Did you receive information today on what you should do if the child has reactions or side effects

to the vaccines?

A. Yes B. No

33. Today, did they write down for you the date of the next vaccination appointment?

A. Yes B. No

34. Are you satisfied with the service provided today?

A. Yes B. No **SKIP TO QUESTION 36**

0

35. Why were you satisfied with the service? (CHECK ALL THAT APPLY)

A. Immediate attention **SKIP TO QUESTION 37**

B. Friendly treatment by staff **SKIP TO QUESTION 37**

C. The necessary vaccines and supplies were available **SKIP TO QUESTION 37**

D. Other Specify: __________ **SKIP TO QUESTION 37**

36. If not satisfied why were you NOT satisfied? (CHECK ALL THAT APPLY)

A. Had to wait a long time

B. The staff was discourteous

C. The language that the health workers use is not clear

D. They did not explain what vaccines they had given the child

D. The necessary vaccines or supplies were not available

F. Other Specify: __________

37a. Have you ever been asked to pay for vaccines given to a child?

A. Yes B. No

37b. Have you ever been asked to pay for a health card/passport for a child?

A. Yes B. No

38. Could you tell me the purpose of vaccines? **(CHECK ALL THAT APPLY) Please DO NOT read out the options**

A. To prevent diseases

B. So children will grow up healthy

C. To cure/heal diseases

C. They don't do any good

D. Not sure what they are for

E. Other Specify: __________

39. Do you think your child could get diseases if you don't vaccinate him/her?

A. Yes B. No

40. What suggestions do you have to improve vaccination services? **(CHECK ALL THAT APPLY)**

A. There should be more vaccination personnel

B. There should be less waiting time

C. Hours and days when vaccination services are available should not be limited

D. Vaccination should remain free

E. The treatment of the public, and of the children being vaccinated, should be friendlier

F. Vaccines should always be in stock

G. They should provide information on the vaccines that are being given, on the diseases that they prevent, and on the reactions that they produce.

H. More outreach services

I. Other Specify: ___________________

J. None

K. Don't know

**Interviewer:** Thank the interviewee and note the time when the interview ended. Read the following statement:

**"Remember that vaccination is a right for all people. Demand this right and remember to bring your**

**child's vaccination card to the health facility each time you visit the center for any reason."**

**Interviewer's remarks:** ____________________________________________________________________

_______________________________________________________________________________________

_______________________________________________________________________________________

**Appendix 4: Key informant interview (KII) guide for health workers/vaccinators and managers**

Good morning/afternoon. My name is _______________________________ and I would like to respectfully ask for your help in answering the questions in this interview. Our goal is to help improve the vaccination program in general. I know you are busy, so we will interview you for only a few minutes. Your participation is completely voluntary and anonymous. Would you be willing to answer these questions?

Thank you very much.

**Date of interview** Day |__|__| Month |___|___| Year |___|___| Interview start time:________________

Name of interviewer: __________________ Supervisor: _______________

Name of health facility/Woreda: __________________

Region __________________ Zone ___________________ Woreda _________________

**Opening questions:**

1. What are some of the health problems that affect children you see at this facility?

2. How are children protected from being affected by these health problems/diseases? (Probe for individual health workers’ roles; If vaccination is not mentioned, ask: What about vaccination?)

1**Key questions: Vaccination services**

1. What can you tell us about vaccination services in this health facility? a. Probe for levels of satisfaction among clients with the vaccination services you are providing; b. Probe for perceptions regarding the vaccination program (e.g. Frequency of sessions, coverage, dropout. etc.)
2. What are some challenges to delivering vaccination services at this health facility? Probe: Logistics, vaccines, human resource, etc.
3. In your opinion, what are some of the ways measles vaccination services can be improved?

**Key questions: Vaccination compliance**

1. In Ethiopia, as you are aware, the national program sets a vaccine schedule. How would you describe compliance with the vaccination schedules in this community? a. Probe for proportion of children that receive all their recommended vaccines on time; b. Probe for reasons why some of the children DO NOT receive all their vaccines at the appropriate time?

7. In some other health facilities, we have been told that there are circumstances when children that come to the facility are not vaccinated. Can you tell me the circumstances when you, or other staff, would not vaccinate a child in this clinic?

a. Probe for contraindications: over age, vial doses, vaccination days, no vaccines, etc.

8. What are your suggestions for helping children to catch up with their vaccinations, if needed?

**Key questions: Missed opportunities**

9. Some children who may not be up-to-date on their vaccinations may visit a health facility for a variety of reasons (immunization, nutrition, treatment of other ailments, accompanying an adult to the clinic/hospital) and may leave without receiving any immunizations. What is your experience with such children at this health facility?

a. Probe: How can they be made to receive the vaccines they are eligible for when they visit the clinic?

10. What strategies, if any, can this health facility employ to improve the number of children receiving all of their recommended vaccinations on time?

a. Probe for ideas or strategies that other critical actors/entities can employ

11. In your opinion, what are the possible barriers to implementing any of these interventions to reduce missed opportunities?

a. Probe for workable solutions to any barriers that have been mentioned

**Closing questions**

12. Are there any additional suggestions/ideas you would like to share at this time? Anything else to add?
