# Supplementary material for: Challenges and enablers in measles vaccination implementation in Ethiopia: Insights from a qualitative study
Source: PLOS Glob Public Health. 2025 Jul 7;5(7):e0004859. doi: 10.1371/journal.pgph.0004859 (PMC12233235; doi:10.1371/journal.pgph.0004859)
Supplement: Attachment — Submitted filename: Report on challenges and enablers in measles vaccination.docx [file pgph.0004859.s002.docx]

Dear Editor-in- Chief,

I have provided my comments on the manuscript, PGPH-D-24-02245R1 entitled, **“Challenges and enablers in measles vaccination implementation in Ethiopia; insights from a qualitative study”**

This manuscript presents challenges and enablers in measles vaccination implementation in Ethopia; insights from a qualitative study. The manuscript is generally written and clearly presented.

The authors have made all the necessary changes.

**Recommendation:** The paper should be accepted

Thank you.

Yours sincerely,

**Olaoye A. Titilayo PhD**

**Title:** The title should be change to reflect the study area. Suggested title: Factors associated with tobacco consumption among in-school adolescents in Nepal

**Abstract**

Line 2 on the abstract should not start with although since the consumption of tobacco among the adolescents in Nepal is still a problem.

Line 4: the objective of the paper should be written in past tense since authors were reporting their findings.

Line 5: the design should simply be written as cross-section design or survey design.

Line 7; the authors should change probability proportional to proportional stratified sampling technique.

Line 8 instead of stating that the sample size was 235 but only 225 were included in analysis, the authors should state that the response rate was 95.7%.

Line 9: the authors need to include that the questionnaire was validated.

Line 12: the statistics value to show the association should be included in this abstract.

Line 15: the recommendation should be change to be all encompassing not just focusing on the girls and who should be responsible for the recommendation made has to be specified.

Line 17 Keywords- factor should change as keywords it can be replaced with parent consumption.

**Introduction**

Line 19 and 26 is stating the same thing on the number of death attributed to tobacco. The authors should be mindful of repeating the same thing.

The author should clearly state the gap and the uniqueness of the study as the factors were already reported by a study as noted in line 62.

**Methods**

Line 75 the authors should state that the study employed a cross-sectional design.

Line 102, the authors should explain the basis for different non-response rate.

Line 103: the authors should explain in details the sampling technique.

How many boys were selected per schools and how many girls? It will be best to have a table to show this distribution and how did they select the students that fill the questionnaire.

Line 114: The authors should include where the pretest of instrument was done and the type of validation that was done in details.

**Results**

**Line 155:** the authors should create a table that shows boys and girls separate instead of lumping them together.

Line 156: the authors should use a binary logistic regression, Chi-square is not sufficient.

Line 256: the recommendation is weak and it should be all encompassing.
